# Exploring hospital resilience protective or risk factors: lessons for future disaster response efforts

**DOI:** 10.3389/fpubh.2024.1378257

**Published:** 2024-03-27

**Authors:** Wenwen Shi, Rujie Chen, Kuan Wang, Yixin Wang, Li Gui

**Affiliations:** ^1^Department of Emergency Nursing, School of Nursing, Navy Military Medical University, Shanghai, China; ^2^905th Hospital of the PLA Navy, Shanghai, China; ^3^School of Nursing, Navy Military Medical University, Shanghai, China

**Keywords:** public health, infectious diseases, hospital resilience, qualitative research, hospital staff

## Abstract

**Background:**

Hospital resilience is essential in responding to disasters, but current research focuses mainly on frameworks and models rather than the protection of resilience and analysis of risk factors during public health emergencies. This study aims to examine the development of resilience in Chinese frontline hospitals during the initial COVID-19 outbreak in 2020, providing insights for future disaster response efforts.

**Objectives:**

We conducted interviews with 26 hospital staff members who were involved in the initial response to the COVID-19 outbreak in China. We used a semi-structured interview approach and employed purposive sampling and snowball sampling techniques. The interview outline was guided by the ‘Action Framework’ proposed by the World Health Organization (WHO) for responding to infectious disease emergencies. This framework includes dimensions such as command, surveillance, risk communication, medical response, and public health response. We analyzed the collected data using Colaizzi’s seven-step data analysis method and the template analysis method.

**Results:**

WHO’s ‘action framework’ effectively highlights the factors that contribute to hospital resilience. While medical response, including the availability of materials and facilities, the use of information technology, and the capacity for infectious disease diagnosis and treatment, remains crucial, other important aspects include awareness and beliefs about infections, treatment experience, interdisciplinary collaboration, and more. Additionally, it is essential to establish an intelligent command system, foster trusting partnerships between teams, improve monitoring capabilities for infectious disease agents, enhance risk communication through information synchronization and transparency, strengthen infection control planning, and improve environmental disinfection capabilities for effective public health emergency response. These contradictions significantly impact the enhancement of hospital resilience in dealing with major infectious disease outbreaks.

**Conclusion:**

In responding to sudden major infectious diseases, hospitals play a vital role within the healthcare system. Enhancing hospital resilience involves more than just improving treatment capabilities. It also requires effective command coordination at the hospital level, infection control planning, and the deployment of intelligent equipment. Additionally, planning for effective communication and coordination between hospitals, communities, and the national healthcare system can further enhance hospital resilience.

## 1 Introduction

With the continuous expansion and deepening of human activities in the natural environment, the contact and interaction between various organisms and humans have increased. This has led to a higher likelihood of unknown infectious disease pathogens invading the human body and causing mass transmission. The insufficient understanding of the pathogenic characteristics, epidemic patterns, and treatment methods of newly emerging infectious diseases has made it challenging to control such events, posing a significant threat and challenge to human health and social stability (1, 2). The COVID-19 pandemic in 2019 serves as a prime example, revealing the global response capabilities to major infectious diseases and emphasizing the importance of building resilient healthcare systems (3). Hospitals, as a crucial component of the healthcare system, play a vital role in epidemic response and serve as the primary point of contact for communities during emergencies. Therefore, ensuring the stability and resilience of hospitals in the face of major infectious diseases is essential (4). Scholars have proposed the concept framework of hospital resilience for disaster response, which includes hospital safety, disaster preparedness and resources, continuous basic medical services, and recovery and adaptation as the four key elements (5). Additionally, some scholars have studied the development of hospital resilience after the Nepal earthquake (6). However, literature specifically focusing on hospital resilience related to biosafety was mainly published after 2019 and is predominantly centered around COVID-19 (7). Despite the increasing attention given to hospital resilience and the gradual enrichment of its concept, the numerous conceptual definitions and model constructions make it challenging to provide practical guidance for hospitals at the operational level. Furthermore, there is a lack of empirical research on the actual participation of frontline hospitals in major infectious disease emergencies.

The concept of resilience has undergone three stages of development. Initially, resilience was used in mechanical research to describe the strength and ductility of materials, referring to their ability to resist or absorb external forces. In the second stage, resilience was seen as the capacity of a system to withstand external disturbances while maintaining its basic function, structure, identity, and feedback (8). The third stage was influenced by Bronfenbrenner’s bioecological theory, which highlights the interdependence between individual development and the environment they are in (9). This theory has contributed to the understanding of resilience in ecology, suggesting that a system’s resilience at a specific level is influenced by the dynamics and states of other levels, resulting in a complex interplay of resilience (10). In this stage, the concept of resilience in systems has evolved from solely focusing on the system’s ability to resist external disturbances and return to its original state or function, to recognizing the mutual influence of various elements within systems, which continuously drive adaptability, reorganization, and development.

Hospitals, like any other systems, are influenced by community management capabilities, healthcare system policies, and various other factors (11, 12). In particular, hospitals can be considered as microsystems, the communities they are located in as mesosystems, other hospitals and communities they do not directly interact with as ecosystems, and health management departments as macrosystems. These macrosystems have an impact on hospitals through their policies and institutions. Our team is currently researching this area. However, the true role of these different systems is reflected in the adaptive capacity of hospitals. After being affected, hospitals will activate and mobilize protective factors to maintain their stability. The interaction between risk factors and protective factors gives rise to four different states: imbalance, obstruction, recovery, and activation. This process is also known as the process model of resilience (13). When hospitals respond to major infectious diseases such as COVID-19, there are factors that influence their response to such events, which can be presented as protective or risk factors.

The process model of resilience includes the “Pressure-State-Response” proposed by Rapport and Friend, which vividly describes the logical relationship between the causes of problems, the changes caused by the problems, and the resulting states and responses through three stages: pressure, state, and response (14). Based on the adaptive cycle’s chaotic model, it is believed that systems are in a state of dynamic change and constantly go through four stages of development: growth or development stage, conservation stage, release or creative destruction stage, and reorganization stage. In the field of disasters, Fink’s life cycle theory is often adopted, which divides the response cycle of emergencies into the latent period, onset period, spread period, and decline period. However, the above models have limitations when applied to the response to infectious disease emergencies. In this study, we explore the generation of protective or risk factors in the five stages of hospital response to infectious diseases: command, surveillance, communication, health care response, and public health intervention, based on the ‘Framework for Action’ proposed by the WHO for responding to infectious disease emergencies (15). This will provide practical references for future hospital responses to major infectious diseases.

## 2 Methods

### 2.1 Study setting

On December 31, 2019, the World Health Organization (WHO) reported cases of a new type of pneumonia caused by a novel coronavirus, which is currently named 2019-nCoV (16). Subsequently, a pandemic outbreak occurred, but it is currently under effective control. Our research specifically examines the early stages of the pandemic, focusing on the direct involvement of healthcare workers in hospitals in Wuhan, China in the initial response to the infectious disease. The experiences and contributions of these healthcare workers serve as valuable primary data for our research on the infectious disease response.

### 2.2 Data collection

**Figure 1:**
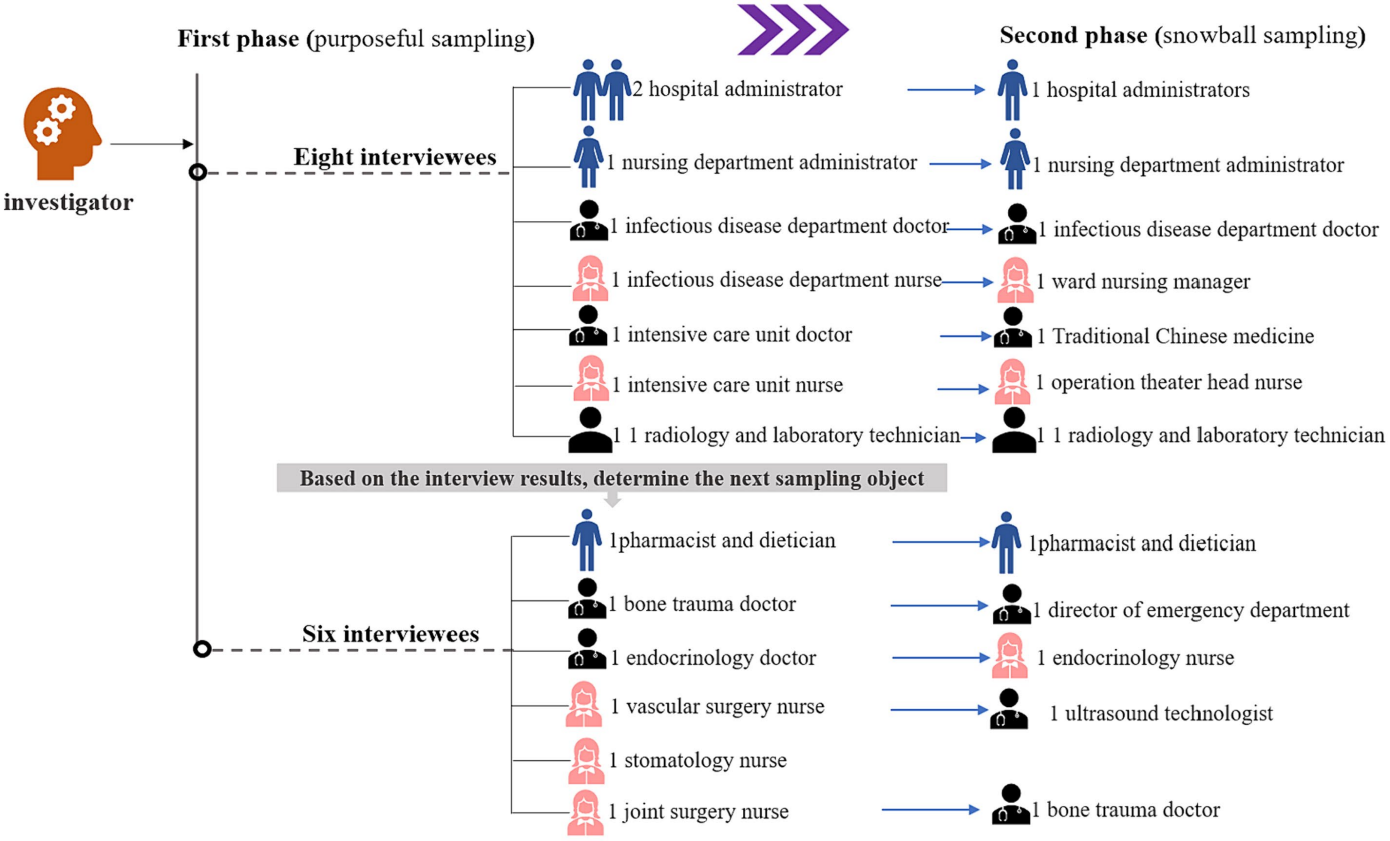
The sampling process (purposeful sampling and snowball sampling).

The operation of hospitals relies on the collaboration of different departments. Therefore, it is essential to analyze the protective or risk factors that impact the resilience of hospitals from the perspective of various positions of hospital workers. This study employed purposive sampling and snowball sampling principles to recruit the initial group of medical staff (see Figure 1), hospital administrators, and other personnel from our affiliated hospital who actively participated in the prevention and control of the Wuhan epidemic. A total of 26 interviews were conducted with personnel who had been to Wuhan to provide support before December 31, 2019. These interviews took place between August and December 2022, with the consent of the interviewees, in quiet meeting rooms. The duration of each interview ranged from 40 to 60 min.

Before each interview, the first author introduced the research content and purpose of the research team to the interviewees, obtained their consent again, and then issued an informed consent form. The interviewees read the form, signed it, and filled out a general information report. Simultaneously, a standardized interview outline was provided for reference. The interview usually began with simple questions to help the interviewees relax and encourage them to express their true feelings. For example, they were asked about their profession and the department they worked in at the hospital in Wuhan. The formal interview outline was based on the ‘Action Framework’ for infectious disease outbreaks by the World Health Organization (refer to Table 1). It covered topics such as command, surveillance, risk communication, medical response, public health response, and overall opinions and suggestions regarding their main work at the hospital in Wuhan. With the interviewee’s consent, the interview was recorded using a voice recorder and transcribed verbatim. Notes were also taken during the interview and included in the initial coding process. The interview records were returned to the interviewees for verification, but no changes were required.

**Table 1:** Interview outline.

Category	Specific questions
Content	What is your main job content at the Wuhan Hospital?
Command	What is the superior command department? What does the command include? Which command work have you participated in? Who are the main personnel in charge? Is the command comprehensive? How do you evaluate the command work?
Surveillance	What aspects of information need to be monitored in your work (which clinical information needs to be reported to you)? What information needs to be reported upwards? Which information will affect the command? Is the monitoring information comprehensive? How do you evaluate the monitoring work?
Risk Communication	Which departments do you communicate with regarding risks in your work? What does the communication include? Is the communication information comprehensive? How do you evaluate the risk communication work?
Medical Response	What is the process for receiving patients in the ward? Has the work of patient admission been smooth? How is the situation regarding patient examination, treatment, medication, and nursing? How do you evaluate the medical response work?
Public Health Response	Does your hospital have responsibilities in public health response? How do you carry out your work? How do you evaluate this part of the work?
Overall Impression	In your experience of epidemic prevention, what aspects of the hospital’s response to major infectious disease outbreaks are worth affirming and why? Or what are the issues worth reflecting on (insufficient) and why?

### 2.3 Data analysis

Following guidelines for qualitative data analysis in health services research, we utilized a combination of deductive and inductive thematic coding (17). The coding and discussion of the results were collectively completed by the team. In the specific coding process, a coding team consisting of the corresponding author and two researchers is formed. Firstly, the two researchers strictly conduct data coding and classification in a double-blind manner. The corresponding author then reads the coding and proposes questions and suggestions. Any discrepancies that arise during the coding process are verified and resolved through supplemental background knowledge and group discussions, aiming to reach a consensus. If a consensus cannot be reached, the coding is deleted. Additionally, weekly group meetings are held to report and discuss the coding process, aiming to improve the accuracy of the analysis results.

Initially, we provide an overview of the research findings, outlining the impact of infectious diseases on hospitals and the corresponding changes observed at each stage. By employing a standardized outline that encompasses multiple dimensions, we observed that the interviewees’ responses largely align with the five dimensions defined by the WHO’s action framework: command, surveillance, risk communication, medical response, and public health response. However, certain aspects of the interview content deviate from the five dimensions. To clarify their attribution to a specific dimension, we conducted a structured reanalysis of these elements.

This study opts for manual analysis rather than computer software for the qualitative data analysis, with the aim of identifying protective or risk factors within the foundational dimensions of the model. To achieve this, we employed a template analysis method based on the WHO’s “Behavioral Framework” for responding to outbreaks of infectious diseases, which encompasses five key elements. The data analysis process adheres to Colaizzi’s seven-step method for data analysis: (1) detailed recording and careful reading of interview data. (2) excerpting meaningful statements that match the research phenomenon. (3) summarizing and extracting meaning from the meaningful statements. (4) searching for common concepts or characteristics of meaning to form themes, theme clusters, and categories. (5) connecting the themes to provide a complete narrative of the research phenomenon. (6) stating the essential structure of the phenomenon. (7) returning the obtained results to the interviewees to verify the authenticity of the content. If new data emerged, it was integrated into the comprehensive description. This study adhered to the Consolidated Criteria for Reporting Qualitative Research (COREQ) (18).

Finally, two situations were identified regarding the protective and risk factors of hospital resilience. In most cases, the protective factors (P) and risk factors (R) were interdependent, with a mutually exclusive relationship. As the protective factors (P) increased, the risk factors (R) gradually disappeared. However, in some cases, the protective factors and risk factors were independent of each other. Increasing the protective factors (P) had no impact on the risk factors (R).

## 3 Results

### 3.1 Characteristics of study participants

We conducted interviews with 26 hospital staff members from various functions and demographic backgrounds, as outlined in Table 2. The subsequent interview objects are represented by I1 to I27.

**Table 2:** Characteristics of interviewees.

Characteristics	Number
Profession	
Nurse (Departments: infectious disease, endocrinology, intensive care unit, joint surgery, vascular surgery, stomatology, operation theater, administration)	8
Medical Doctor (Departments: infectious disease, bone trauma, intensive care unit, endocrinology, anaesthesiology department, administration)	7
Administration	5
Pharmacist and dietician	2
Radiology and laboratory technician	2
Ultrasonography lab	1
Traditional Chinese medicine	1
Gender	
Male	15
Female	11
Age Group (at time of the interview)	
20 ~ 30	4
30 ~ 40	13
40 ~ 50	9

Based on the information gathered from these interviews, we identified five dimensions that encompass the protective factors and risk factors contributing to hospital resilience. These dimensions effectively capture the approaches, strategies, and perspectives of the interviewees in addressing major infectious disease outbreaks. Additionally, we observed that the interviewees not only analyzed the response from the hospital’s standpoint but also shared their personal perspectives. Consequently, we have included several personal-level protective factors and/or risk factors in Table 3, building upon the aforementioned five dimensions.

**Table 3:** Overview of hospital resilience protective and risk factors: content analysis based on interview information.

Framework for Action	Protective factors/ Risk factors	Protective factors	Risk factors
Command	Combination of different teams within the same department	Analysis of the disease and formulation of treatment plans Clear division of labor Dynamic adjustment and redistribution of resources	
Surveillance		Timely inventory and replenishment of supplies Health monitoring of medical staff, especially monitoring of infectious symptoms Monitoring of the environment, especially the living area environment Monitoring of public opinion Level of information monitoring Supervision of work quality. Supervision of personal protection Provision of electronic information communication devices	Repetitive monitoring
Risk communication	Smooth transmission Convenience of communication	Infection explanation early warning Information synchronization Information transparency Retrospective analysis	Communication barrier caused by isolation environment
Medical response	Ability of daily diagnosis and treatment services	Special training on infection prevention and control for medical staff Experience in the treatment of infectious diseases Clear patient treatment processes Multidisciplinary treatment plans Telemedicine	
Public health response		Standardized spatial renovation Adequate protective equipment Public health personnel Medical waste disposal	
Individual resilience		Firm goals Stability of families	Excessive protection Fatigue from protection

### 3.2 Protective and risk factors for hospital resilience

#### 3.2.1 Command dimension

During the initial phase of a pandemic outbreak, hospitals often have personnel from various units working together. The amalgamation of different teams within the same department can have both positive and negative effects. On one hand, effective collaboration can result in a synergistic effect that surpasses the individual contributions. On the other hand, lack of familiarity among personnel can hinder coordination and impact the seamless progression of work.

The hospital command system’s command work is built upon a thorough analysis of the epidemic situation. This analysis serves as the basis for formulating appropriate treatment plans and defining roles. Furthermore, hospitals may need to dynamically adjust and control various aspects such as medical staff, departments, and the number of admitted patients to maintain a balance in internal operations, especially when faced with limited medical resources and nursing services (Table 4).

**Table 4:** Representative quotes obtained from participants in the command dimension.

Protective factors/ Risk factors	Quotes obtained from participants
Combination of different teams within the same department	I22“So, like, when we were first getting things ready for the wards, we realized that there were 2 units assigned to one ward. So, you had two small medical teams working together in one ward, you know? And as the commander, it was really tough to give orders and lead them. They did not report to you, because it’s not like you have authority over other teams, right? You could not really tell them what to do. It was just a frustrating situation, you know?”
Analysis of the disease and formulation of treatment plans	I5: “If I were a team leader, I might first consider the task I am assigned and analyze it. What kind of task is it? What kind of person do I need for it? Then, I might distribute the personnel accordingly. I22: “You need to consider what the task is and if you take this group of people out, you have to think about what kind of tasks I can take on with this group. Because sometimes the task may change after going out, so you also need to do some task analysis.”
Clear division of labor	I21: “One good thing they did was organize things systematically. They would divide us into groups for different tasks. For example, there was a group specifically responsible for admitting patients, another group for collecting nucleic acid samples from patients, and another group specifically assigned to disinfect the hotel. They had dedicated teams for each task.”
Dynamic adjustment and redistribution of resources	I15:“Well, you see, we had so many really sick patients that we had to set up a special unit kind of like an intensive care unit. That unit was in charge of taking care of the most critical cases, but sometimes our ward would have patients who were really, really sick and they needed to be transferred to that unit. The thing is, though, that unit did not have a doctor from the emergency department, but we did. So, they decided to move our emergency department doctor to their team so that they could handle cases in the ward.”

#### 3.2.2 Surveillance dimension

Monitoring plays a crucial role in responding to infectious disease outbreaks, encompassing surveillance, supervision, and various aspects of hospital resilience. It is important to monitor the presence of the virus in the environment, particularly in the living areas of healthcare workers. Additionally, monitoring the usage of supplies and ensuring timely replenishment are also essential protective measures. Furthermore, during the initial stages of a pandemic outbreak, healthcare workers face significant challenges and stress, primarily due to the requirement of wearing protective equipment. Therefore, it becomes imperative to monitor their health closely. It is worth noting that this situation differs from other emergency response scenarios.

In major infectious disease outbreaks, various media platforms become avenues for public assistance. Hospitals actively pay attention to epidemic-related public opinions to gain insights into the public’s medical condition and verify the accuracy of hospital-related information. This proactive approach helps prevent the dissemination of misleading information that may lead to unnecessary panic.

Information monitoring devices can aid hospitals in remotely supervising the quality of work and ensuring that healthcare workers are wearing appropriate protective equipment. However, determining the optimal scale of monitoring poses a challenge. Excessive monitoring within a short timeframe can increase workload and reduce efficiency (Table 5).

**Table 5:** Representative quotes obtained from participants in the surveillance dimension.

Protective factors/ Risk factors	Quotes obtained from participants
Timely inventory and replenishment of supplies	I7: “Regarding epidemic prevention materials, all types of materials are managed by the administrators in charge. At that time, our materials were strictly monitored for outgoing and incoming inventory, including registration procedures, which were strictly implemented. For food-related materials, we also classified and stored them, analyzed them, and closely monitored their conditions. We could determine whether they were in good or bad condition, and their size and model. We were also aware of the movements of these materials.”
Health monitoring of medical staff, especially monitoring of infectious symptoms	I11: “Then we will also assess the situation and psychological state of nurses, for example, to see if they can handle it. At the beginning, when there were not many patients, we would evaluate their condition, their ability to adapt to the work, and their willingness. If they are willing to work in the high-risk areas, we would assign them there. However, if they cannot adapt or feel that they cannot bear the work, we would assign them to work outside.” I15: “Also, after we go back, the head nurse would usually ask if we have any discomfort. Because working inside the protective clothing can be suffocating and uncomfortable at first, there may be situations like shortness of breath and dizziness when we return. But fortunately, it’s not too common, and after working for a few days, we get more adapted to it.”
Monitoring of the environment, especially the living area environment	I6: “There is also environmental monitoring for our accommodation area, such as cross-infection, as well as monitoring of symptoms and body temperature for us team members.” I8: “Monitoring includes not only the area where we remove our protective clothing but also the living area and the wards. They will monitor things that we have come into contact with or may come into contact with to check for any viruses. This monitoring serves the purpose of supervision as well. Moreover, we are aware of blind spots and strive to improve our disinfection work.”
Monitoring of public opinion	I24: “There is a dedicated person to monitor public opinion… It’s quite unique, especially for major infectious disease incidents like this, public opinion monitoring is definitely necessary.”
Level of information monitoring	I9: “We repeatedly emphasize the application for monitoring, so that many people can go through this procedure to monitor the washing and removing of protective clothing by personnel in various areas. Once any non-compliance is detected, it is promptly addressed, which strengthens the supervision.”
Supervision of work quality	I18: “The nursing department also conducts inspections on us, with the sole purpose of ensuring the safety and effectiveness of our work. Because it not only relates to the safety of patients but also to the safety of us healthcare workers ourselves.”
Supervision of personal protection	I10: “Ensuring the personal protective equipment of healthcare workers is qualified is the most important aspect in the prevention and control of infectious diseases. While healthcare workers themselves also attach great importance to this, necessary supervision mechanisms are necessary measures to ensure safety.”

*“We focus on using surveillance in our work. Apart from the red zone, we can keep an eye on the movement of staff in different non-red zones using a program. If we notice any irregularities, we can promptly remind them as we can engage in dialogue and consultation. This has improved our supervision compared to how things were done in the past.”*

#### 3.2.3 Risk communication dimension

During the initial phases of a significant outbreak, the use of protective equipment and quarantine measures can create communication barriers that pose a risk to hospital resilience. To mitigate this, hospitals can implement electronic communication devices, which can enhance resilience. However, the effectiveness of these devices relies on the smooth transmission of information and communication.

Lack of understanding among healthcare workers regarding protective measures and treatment protocols in infectious disease environments, as well as the public’s limited comprehension of the necessity for frequent nucleic acid testing, contribute to the challenges of infection explanation and early warning. To address these issues, hospitals can provide explanations, demonstrations, and education to clarify the reasons for and risks associated with infection.

*“If you just give healthcare workers instructions, they might follow them, but not necessarily do a great job. But if you explain why they should do it, once they grasp the reasons, they can perform their tasks more smoothly and find better solutions to problems. They will also have a stronger sense of initiative to accomplish these tasks.”*

During major infectious disease outbreaks, there is a significant need for healthcare workers and the public to access information about disease transmission and updates on prevention and control measures. It is crucial to ensure that information is acquired and disseminated in a synchronized and transparent manner to enhance the trust of healthcare workers and the public in the hospital.

Healthcare workers involved in treating infectious diseases face various challenges and issues in their work, including treatment plans, infection control, emergencies, and protective procedures. Conducting post-incident reviews and making timely improvements based on the experiences and lessons learned can help prevent the occurrence of risky events (Table 6).

**Table 6:** Representative quotes obtained from participants in the communication dimension.

Protective factors/ Risk factors	Quotes obtained from participants
Smooth transmission	I2: “I think the communication is relatively smooth. For example, the medical department and the nursing department assign dedicated personnel to communicate with me on various matters. They will inform me about everything related to the patient’s admission to discharge.”
Convenience of communication	I7: “Communication with personnel can be quite troublesome. If you do not know someone personally, it becomes difficult. This time, compared to the previous incident in Wuhan, the infection control requirements are higher. When wearing protective clothing, we cannot carry our phones with us. So sometimes, it’s inconvenient to find someone for urgent matters.”
Infection explanation early warning	I23: “Risk identification is crucial. For example, in terms of communication among staff, I told everyone to carry an alcohol swab in their hands while working and use it to wipe surfaces. But they did not understand. I also told them that besides wearing masks, they should disinfect their hands. But they did not understand either. So, I had to address this issue and make everyone realize how important it is. I showed them films related to infectious disease prevention, and immediately everyone understood. They understood the importance of cleaning hands and surfaces.”
Information synchronization	I5: “After the nurse receives the sampling plan sheet in the morning, they will contact the community leader of the neighborhood committee to inquire about the number of people to be sampled today. They will also confirm whether it’s individual sampling or pooled sampling and whether the neighborhood committee has communicated with the residents in advance. If there is no proper communication, they have to wait until the communication is done before proceeding.”
Information transparency	I9: “One thing I think is particularly good about the hospital is that they do not evade issues. Whenever something happens in the hospital, they hold meetings, and online conferences inform us about the current situation. The transparency of information helps us have a clearer understanding of what steps we should take next.”
Retrospective analysis	I9: “At 7 o’clock in the evening, we have a hospital-wide handover session where all department directors and head nurses participate in the group. The hospital’s management, including some functional departments like infection control and pharmacy, also join. We report on the day’s work and discuss any issues that arise. We analyze and record the problems encountered to guide future work and improve treatment efficiency.”
Communication barrier caused by isolation environment	I11: “Because we wear masks and face shields, others cannot hear our voices clearly. It makes communication difficult. We have to speak loudly and shout in order for others to hear us, otherwise, they will not be able to hear us.”

#### 3.2.4 Medical response dimension

Proper training is essential for healthcare workers who are not specialized in handling infectious diseases. Incorporating previous experiences in managing infectious diseases like SARS can help healthcare workers quickly improve their skills. It is important for hospitals to establish clear patient treatment processes to ensure that healthcare workers understand the patient’s access and flow. Multidisciplinary treatment plans, such as traditional Chinese medicine and hyperbaric oxygen chambers, have demonstrated good efficacy in responding to COVID-19 and other similar emergencies.

*“So, we had this traditional Chinese medicine doctor who gave us some Chinese herbal medicine for every unit, and it really helped, especially during the later part of the recovery process. I believe it had a positive impact on rehabilitation, therapy, and overall conditioning.”*

During a pandemic, the diagnostic and treatment capacity of hospitals may be overwhelmed. In such situations, telemedicine can serve as an alternative means to assist the public with medical issues and ensure the smooth operation of hospitals. This is particularly relevant during infectious disease outbreaks (Table 7).

**Table 7:** Representative quotes obtained from participants in the healthcare response dimension.

Protective factors/ Risk factors	Quotes obtained from participants
Ability of daily diagnosis and treatment services	I8: “During the epidemic, the hospital’s capacity for routine diagnosis and treatment is also a crucial factor in testing its resilience. If the hospital has a well-developed telemedicine system and can meet the normal diagnosis and treatment needs during the epidemic, it can also play a positive role in infectious disease prevention and control. However, during this epidemic, we found that the hospital’s telemedicine system was challenged.”
Special training on infection prevention and control for medical staff	I4: “When the epidemic occurred in our hospital, we provided training to medical staff, including knowledge about the epidemic and medical procedures. So, when we were working on the front line, I felt that everyone was competent.”
Experience in the treatment of infectious diseases	I2: “Having a certain foundation is still important. Because I had participated in the treatment of SARS in 2003, my response was quick. After receiving the information, we quickly transformed the vacant floor according to the requirements for respiratory infectious diseases.”
Clear patient treatment processes	I3: “If a patient suddenly experiences changes in their condition, we can mobilize standby medical staff from outside to assist. Each floor of the hospital has an emergency department doctor who is on duty 24 h a day. Once a patient experiences changes in their condition or even sudden symptoms, the doctor from the emergency department will immediately go in and provide treatment.”
Multidisciplinary treatment plans	I14:“So, we had this traditional Chinese medicine doctor who gave us some Chinese herbal medicine for every unit, and it really helped, especially during the later part of the recovery process. I believe it had a positive impact on rehabilitation, therapy, and overall conditioning.”
Telemedicine	I17: “During the epidemic, patients still need health education after discharge. In the past, patients would come to the hospital for this purpose, but during the epidemic, we have conducted online education. This is also thanks to the many online courses we developed earlier, which have been very helpful during the epidemic.”

#### 3.2.5 Public health response dimension

Hospitals can enhance the conditions for treating infectious diseases by remodeling their facilities and installing negative pressure wards, fresh air systems, and other air purification devices. This helps in preventing nosocomial infections. Additionally, hospitals should ensure the availability of personal protective equipment, strictly follow infection control measures, and promptly manage medical waste to prevent further spread and transmission of infectious diseases. Public health professionals play a crucial role in providing solutions and support for hospitals’ scientific public health response (Table 8).

**Table 8:** Representative quotes obtained from participants in the public health response dimension.

Protective factors/ Risk factors	Quotes obtained from participants
Standardized spatial renovation	I10: “First and foremost is safety. The ward I am currently in used to be the obstetrics and gynecology department, but now it needs to be transformed into an infectious disease department. This requires the establishment of isolation areas (the first isolation area and the second isolation area), as well as improvements to some routine corridors to make them suitable for infectious disease treatment.”
Adequate protective equipment	I18: “I believe that the first priority is to ensure the safety of healthcare workers. If healthcare workers are exposed to hazardous environments and cannot guarantee their own health, the quality of patient care will also decline. Therefore, hospitals must first provide an adequate supply of protective equipment to ensure the safety of healthcare workers.”
Public health personnel	I5: “It is necessary to reserve some emergency health medical teams to deal with unexpected situations, and hospitals should also provide professional training and regulations for these teams to ensure quality work.” I14: “The importance given to the infectious disease department may be relatively low during normal times, but I believe that proper staffing is necessary. Even if the hospital does not have sufficient staffing, the public health system should strengthen talent reserves to guide healthcare workers in proper protection and treatment during major outbreaks of infectious diseases.”
Medical waste disposal	I7: “Regarding waste management, we follow the practice of treating all waste generated in our own rooms as medical waste. This includes using double-layered yellow garbage bags with a goose-neck tie, which are then disinfected at the door using a 1,000 mg/L effective chlorine solution. Designated personnel will collect and transport the waste for centralized management and disposal.”

#### 3.2.6 Individual level

The resilience of healthcare workers is crucial for maintaining the resilience of hospitals. In the early stages of a pandemic outbreak, healthcare workers not only face the risk of infection but also experience significant work pressure, all while worrying about the safety of their families. Despite these challenges, they express pride and gratitude for their commitment to frontline work. The support from their families provides them with courage and strengthens their determination to overcome the epidemic. However, it is important to note that some healthcare workers may underestimate the severity of the outbreak, leading to a lax approach toward protective measures, which can further increase their psychological stress.

*“I never expected something like this to happen to us, and all of a sudden, we had to deal with it without being mentally prepared.”*

*“There is excessive protection in some cases, and this situation is very common, where people wear three or four layers of masks.”*

## 4 Discussion

Our study aims to investigate the actual experiences and emotions of healthcare workers in hospitals during the initial phases of a significant pandemic outbreak. Following the World Health Organization’s ‘Action Framework’ for responding to infectious diseases, we examine the factors that contribute to hospital resilience at each stage, both in terms of protection and risk. In contrast to current hospital resilience frameworks or models, our study offers more precise operational recommendations for enhancing hospital resilience.

At the onset of the COVID-19 pandemic, the concept of hospital resilience was relatively underexplored, primarily with a focus on disaster response. The Multidisciplinary Center for Earthquake Engineering Research (MCEER) in the United States proposed the classic 4R resilience theory, which subsequently served as a theoretical framework for subsequent resilience studies (19). With the outbreak of the pandemic, hospital resilience gained attention; however, most literature examined hospital resilience within the broader healthcare system rather than as a distinct entity. In our study, we specifically concentrate on the hospital system and conduct interviews with healthcare workers from various positions to gain insights into their experiences during the early stages of the pandemic. Building upon Richardson et al.’s resilience process model, we place emphasis on exploring protective and risk factors in establishing hospital resilience and offer practical operational guidance (13).

In this study, healthcare workers were temporarily assigned from different units, which is a common model in emergency response to infectious diseases (20). This model facilitates collaboration among healthcare professionals from various disciplines to deliver comprehensive treatment. However, it also presents challenges for command and management personnel, as the effectiveness of management directly affects the development of hospital resilience. Therefore, management should be guided by timely analysis of the pandemic situation, the development of scientific treatment plans, and the rational allocation of personnel.

Resilience development necessitates ongoing adaptation (21). Numerous studies have highlighted the dynamic nature of resilience (22, 23). Our findings indicate that responding to major infectious disease outbreaks, particularly in their early stages, demands continuous adjustment and optimization of response plans. This represents a significant departure from the routine operations of hospitals, which have well-established systems in place. However, during major infectious disease outbreaks, the situation is often characterized by uncertainty and unknown factors. As a result, management personnel must adjust resources based on the trajectory of the disease, necessitating regular review. Experienced personnel, including those who have dealt with previous infectious disease outbreaks, as well as professionals in public health, play a crucial role in guiding this process.

We have also identified several unique aspects related to major infectious disease outbreaks. One crucial aspect is the continuous monitoring of healthcare workers’ health, particularly for symptoms of infection, to ensure their ability to work under normal conditions. Previous studies have highlighted the dual burden faced by healthcare workers during disaster response, as they juggle family responsibilities and hospital duties (24, 25). However, our study revealed that healthcare workers expressed positive coping strategies, such as having clear goals and receiving support from their families. This reflects the unity among the people during that national crisis. Given the extensive scope and rapid spread of the pandemic, both the general public and healthcare workers paid close attention to news updates, and the timely and transparent dissemination of information became crucial factors influencing the resilience of hospitals. The increased demand for online consultations by the public during the pandemic underscored the importance of continuous routine healthcare in bolstering hospital resilience. Consequently, telemedicine emerged as a protective factor in the development of hospital resilience.

Quality control in response to infectious diseases should not be neglected. Regular inspections by internal and external stakeholders, such as hospitals, health authorities, and other relevant organizations, are necessary to evaluate the effectiveness of the hospital’s epidemic prevention and treatment measures, identify potential risks, and implement necessary improvements. Information monitoring devices within hospitals can provide a convenient means of monitoring, enhancing efficiency, and minimizing personnel movement, thereby preventing cross-infection. However, it is important to strike a balance as excessive monitoring during an epidemic can significantly burden healthcare workers.

The public health response is a crucial factor that distinguishes major infectious disease outbreaks from other disasters. In order to prevent further spread and transmission, hospitals should prioritize the provision of personal protective equipment and diligently enforce infection control measures. These measures include thorough surface cleaning and disinfection, as well as proper management of medical waste, to prevent nosocomial infections (26). The unique characteristics of protective equipment present challenges in communication among healthcare workers, as restrictions may limit verbal communication and the use of mobile phones. Consequently, there has been a focus on developing and enhancing information communication devices.

## 5 Limitations

Although our selection of interviewees was diverse, it is possible that we overlooked certain roles, such as hospital directors and personnel from disease control centers, that could have provided valuable insights and enriched our analysis. Additionally, it is important to note that the protective and risk factors for hospital resilience identified in this study may be influenced by the broader community, healthcare system, and other hospitals, which were not included in our research. Therefore, it is crucial for future studies to thoroughly investigate these factors to gain a more comprehensive understanding.

## 6 Conclusion

This research paper has shed light on the critical role of resilient hospitals in effectively managing major infectious disease outbreaks. By adhering to the World Health Organization’s infectious disease response framework and conducting interviews with frontline healthcare workers, this study has identified protective and risk factors for hospital resilience at each stage. The findings of this research offer valuable guidance to hospitals in their efforts to enhance resilience in future actions. Moreover, this study reinforces the significance of individual well-being in facilitating sustained work and resilience development within hospitals, aligning with prior research. Ultimately, this research paper contributes to the field by presenting a comprehensive approach to addressing the challenges posed by infectious disease outbreaks and fostering hospital resilience.

## Data availability statement

The raw data supporting the conclusions of this article will be made available by the authors, without undue reservation.

## Ethics statement

The studies involving humans were approved by the Ethics Committee of the Navy Military Medical University, the author’s affiliated institution. The studies were conducted in accordance with the local legislation and institutional requirements. The participants provided their written informed consent to participate in this study.

## Author contributions

WS: Conceptualization, Data curation, Formal analysis, Writing – original draft, Writing – review & editing. RC: Conceptualization, Data curation, Formal analysis, Investigation, Writing – original draft. KW: Data curation, Investigation, Writing – original draft. YW: Writing – review & editing. LG: Conceptualization, Formal analysis, Funding acquisition, Project administration, Resources, Supervision, Writing – review & editing.
